# Bullying and the Effectiveness of a Health Education Program Among Female Middle School Students in Riyadh, Saudi Arabia: An Application of Social Cognitive Theory

**DOI:** 10.7759/cureus.85269

**Published:** 2025-06-03

**Authors:** Amani A Alghamdi, Khlood H Almutairi, Nada A Bin Ateeq, Sara A Alsuhaibani

**Affiliations:** 1 Health Education, Princess Nourah Bint Abdulrahman University, Riyadh, SAU; 2 Department of Health Science, College of Health and Rehabilitation Science, Princess Nourah Bint Abdulrahman University, Riyadh, SAU

**Keywords:** adolescents, attitude, bullying, female middle school students, health education program, saudi arabia, social cognitive theory, victimization

## Abstract

Bullying is a significant issue that poses various health concerns, particularly in school environments. The study aimed to determine bullying practices and assess the health education program's effectiveness in changing bullying attitudes among female middle school students using the social cognitive theory (SCT). An interventional one-group pretest/post-test design study was conducted among 304 female middle school students in Riyadh. The primary outcome was the change in students’ attitudes toward bullying after the intervention. This study revealed that 97.7% (n=297) of the students did not consider themselves bullies, and 70.7% (n=215) had a neutral attitude toward bullying. Regarding the SCT conceptual elements, a relationship was found between bullying and bullying's attitude (p = 0.000); there was also a significant association between bullying and the social environment (p = 0.025). The effectiveness of the health education program was highly significant (p=0.000, Z=-4.725). Based on the current study findings, the majority of the participants reported themselves as not bullies, and their attitude toward bullying was neutral. Conversely, there was no significant relationship between attitude toward bullying and the social environment. Lastly, the health education program was very successful. Thus, building a curriculum is necessary to promote anti-bullying attitudes in schools, besides carrying out further qualitative theory-based studies to explore the factors behind the pro-bullying attitude in Saudi Arabia.

## Introduction

Bullying has become an ongoing topic of interest and has cultivated major concerns in the last couple of decades [1]. Also known as “victimisation,” bullying is defined as the misuse of power [2]. For a person to be identified as a bully, certain characteristics have to be presented: an imbalance in power between the bully and victim(s), the act of bullying has to be done with repeated exposure from the bully to the victim over time, and the intent to inflict harm on the victim. [3]. A study suggested that bullies are at high risk for several short- and long-term consequences, such as dropping out of school and engaging in criminal actions [4].

The bullying cycle involves different parties, including victims, bully-victims, bullies, and bystanders. Bullies who have never bullied others are known as victims [5]. They are more likely to become depressed and anxious [6]; studies indicated that victims are more likely to attempt suicide and have suicidal thoughts compared to those who have never faced bullying [7]. Bully-victims are individuals who have been bullied and subsequently become bullies, whereas bullies are those who harm others without having experienced bullying themselves. Bully-victims are more likely to face psychosomatic symptoms such as abdominal pain, headache, and decreased appetite than those who are never involved in bullying incidents [6].

In addition, individuals who witness bullying can significantly influence bullying behaviour in either a negative or positive way [8]. These witnesses are referred to as bystanders and can take on four different roles in the bullying cycle: the defender (e.g., helping the victim), the outsider (e.g., avoiding participation in the bullying situation), the supporter (e.g., laughing and clapping), and the assistant (e.g., supporting the bully) [9]. A study reported that observing bullying can be associated with mental health issues [10].

There are two types of bullying: direct and indirect [11]. Direct bullying, as the name suggests, occurs when the bully directly confronts the victim. Bullying can be physical, like tripping, hitting, or stealing [12]. Verbal bullying can also occur through taunting, mocking, or name-calling [13]. On the other hand, indirect bullying occurs when the victim is excluded intentionally from a social group [13], gossiped or rumoured about behind his/her back [13], or even cyberbullied, where bullying messages are sent via the internet [9]. Moreover, the incidence of either type of bullying differs between males to females. Studies have indicated that females favour verbal and relational bullying more than any other form of bullying. On the other hand, males typically engage in physical bullying [14].

Bullying exists in places with inadequate supervision, including school, the workplace, or even home [15]. However, bullying in school is a global phenomenon, as it occurs with most adolescents internationally [16]. As bullying behavior is a cognitive-behavioral phenomenon related to attitude, it is indicated that there is a need to apply social cognitive theory to health education interventions [17]. Therefore, to fill this gap, this was a theory-based study targeting bullying attitudes in adolescents from female middle schools.

Research question

How does a health education program impact middle school adolescents' attitudes towards bullying and their likelihood to engage in such behaviour?

Objectives

The aim of this study is to measure self-perception of bullying practice among middle school adolescents in Riyadh, KSA. It also seeks to assess the attitude toward bullying among middle school adolescents in Riyadh, to identify the effect of the social environment on bullying, and to identify the effect of internal stimulation on bullying among middle school adolescents.

## Materials and methods

Study design 

The study was an interventional one-group pretest/post-test design conducted through three phases. The first phase was assessing self-perception of bullying practice and the students' attitude. The second phase involved implementing a health education program. The last phase was reassessing students' attitudes toward bullying.

Place and duration 

The study was carried out in four schools, between private and public female middle schools in Riyadh, and it lasted for three months, from January until April of 2019.

Participants

The study's population included adolescent students in both private and public female middle schools. The study included students who were registered at least for one semester in the school because part of the questionnaire investigates the school's environment; therefore, a semester was considered the minimum timeframe to witness or experience any aggressive behaviour.

Sampling

A multi-staged technique was used to recruit participants; the first stage was stratifying middle schools in Riyadh into private and public schools, then from each stratum, two schools were selected by a simple random sampling lottery, depending on a list from the Ministry of Education (MOE) website. Finally, from each selected school, classes were chosen based on a lottery and taken as a cluster. The sample size was 152, however, we increased it to 304 to overcome the missing information.

OpenEpi website version 3.01 (Dean et al., 2013) was used to calculate the sample size depending on three parameters; the unexposed to the Health Education Program (HEP) was 31% (Al-Qahtani, 2013), the exposed to the HEP was calculated to be 11%, and the confidence interval was 95%. By using the proportional allocation calculation, the total sample size of 304 was divided into 239 students for public schools and 65 students for private schools.

Proportional allocation: $nh=Nh/&delta;N&times;n$

Data collection tools

This study used a self-reported standard questionnaire from the Centers for Disease Control and Prevention (CDC) [18] to gather necessary information about bullying and attitudes towards it based on social cognitive theory.

A standard questionnaire from the CDC was provided, composed of four sections (Exposure to Violence and Violent Behaviour Checklist, Adolescent Peer Relations Instrument, and Bully survey) in addition to the sociodemographic section. However, the last section was distributed to the participants twice before and after the intervention. Further, it was translated from English into the Arabic language by the "Sultan for Certified Translation" centre.

The first section included 11 questions, which requested information on the participant’s nickname, birthdate, grade, educational level of parents, the number of brothers and sisters, the student's order between her siblings, and her parents' marital status. 

The second section was a 4-point Likert scale (1=never, and 4=often), and the items were divided into three domains at school, family(home), and community (neighborhood), which were considered as the social environment. The highest scores reflected more bullying experience. However, the social environment was divided into two categories: any participant who scored an average of 32 to 80 had a good environment, and any participant who scored an average of 80.1 to 128 indicated that she had a bad environment.

The third section was used to measure the verbal, social, and physical bullying using a 6-point Likert scale (1=never, 2= sometimes, 3= once or twice a month, 4= once a week, 5= several times a week, and 6= every day). The cutoff scores were as follows: any participant who scored 18 to 63 as a total was considered to be “never bullied others,” and any participants who scored 63.1 to 108 were considered as “bullies.” Further, for the subscales, any participant who scored an average of 6 to 21 points was considered to have never bullied, while a score of 21.1 to 36 points indicated the participant was considered a bully.

The fourth section was used to assess the attitude toward bullying, as well as to measure internal stimulation as an element of social cognitive theory. The questionnaire was given to the participants pre- and post-intervention to assess the effectiveness of the intervention. The survey was a 5-point Likert scale (1= totally false, 2=sort of false, 3=both true and false, 4= sort of true, and 5= totally true). The range for the scale was (15-80), where a score of 55.1 and more indicated a positive pro-bullying attitude, scores of 35.1 to 55 indicated a neutral pro-bullying attitude, and scores of 16 to 35 indicated a negative pro-bullying attitude

Health education program (HEP)

The intervention for this study was an audio-visual HEP, which continued for a week for each school between the field and social network channels. In each school, the program included a lecture to introduce the topic of the study, followed by videos about bullying. The program was concluded by activities that mainly focused on presenting case scenarios and engaging the audience by asking them to vote if they agree/disagree or do not know, followed by discussing their point of view and arguments. Additionally, there were posters hung on the walls of the classes and hallways of schools. Multiple flyers about bullying were distributed to the students. The program also displayed different messages through social media applications (X (formerly Twitter) and Snapchat) to enhance the anti-bullying attitude. After a week of implementing the program, post-questionnaires were distributed to the students.

Primary outcome

The primary outcome of this study was the change in students’ attitudes toward bullying after implementing the health education program, as measured by pre- and post-intervention responses to a validated attitude scale based on the Social Cognitive Theory framework.

Reliability

We conducted a pilot test by recruiting 10 participants before distributing the questionnaire to the main sample. The questionnaire’s reliability was tested by Cronbach’s alpha (α), which is used to test the questionnaire's internal consistency. Cronbach’s alpha (α) of the Exposure to Violence and Violent Behavior Checklist was (α =0.834), for the Adolescent Peer Relations Instrument it was (α=0.861) and for the bully survey after the removal of item (I) "I can understand why someone would bully other kids" it increased to be (α =0.726) after it was (α = 0.678).

Statistical analysis

The data was conducted, coded, and tabulated using Statistical Package for Social Science (SPSS) version 23 (IBM Corp., Armonk, NY), and alpha error was set at 0.05. The data was presented in inferential and descriptive tables in frequencies and figures. Socio-demographic characteristics were displayed in a frequency table. Fisher's exact test was used to analyse the association between bullying practice and social environment, due to the small, expected cell counts in some contingency tables, which makes it more appropriate than the chi-square test in such cases. Chi-square test was used to analyse the association between bullying practice and attitude toward bullying, as both variables were categorical and met the assumptions for this test. Spearman’s correlation was employed to assess the relationship between students’ attitudes toward bullying and their perceived social environment, as both variables were ordinal and the data did not meet the assumptions for parametric correlation (e.g., Pearson’s). Finally, the Wilcoxon signed-rank test was used to compare students’ attitude scores before and after the health education program because the data were paired, non-normally distributed, and measured at an ordinal level. These statistical methods were chosen to ensure appropriate handling of the data type and distribution, enhancing the reliability of the findings. Additionally, the prevalence of bullying practice, bullying types, and students' attitudes toward bullying were detected and presented in percentages. Additionally, the prevalence of bullying practice, bullying types, and students' attitudes toward bullying were detected and presented in percentages.

## Results

Descriptive statistics

Table 1 demonstrates the sociodemographic characteristics of the sample. The recruited students from public schools were 78.3% (n=238), whereas 21.7% (n=66) were from private schools, and almost all students were evenly distributed within middle school grades.

**Table 1:** Sociodemographic characteristics of female's middle school students in Riyadh

Variable	Categories	n	(%)
School type			
	Public	238	78.3
	Private	66	21.7
Educational level			
	First level	75	33.5
	Second level	74	33
	Third level	75	33.5
Previous semester grade			
	Pass	7	3
	Good	19	8.2
	Very good	82	35.2
	Excellent	125	53.6
Mother’s educational level			
	Not educated	10	3.4
	Primary school	25	8.5
	Middle school	32	10.9
	High school	81	27.6
	Bachelor's degree	99	33.7
	Higher than bachelor’s degree	47	16
Father’s educational level			
	Not educated	4	1.4
	Primary school	13	4.5
	Middle school	26	9
	High school	92	31.7
	Bachelor's degree	73	25.2
	Higher than bachelor degree	82	28.3
Siblings (brothers)			
	Has no brothers	25	8.2
	One brother	65	21.4
	Two brothers	93	30.6
	Three brothers	63	20.7
	Four brothers	22	7.2
	Five brothers	20	6.6
	Six brothers or more	16	5.3
Siblings (sisters)			
	Has no sisters	35	11.5
	One sister	62	20.4
	Two sisters	70	23
	Three sisters	64	21.1
	Four sisters	40	13.2
	Five sisters	18	5.9
	Six sisters or more	15	4.9
Birth order			
	First	85	28.1
	Second	73	24.2
	Third	50	16.6
	Fourth	26	8.6
	Fifth	31	10.3
	Six	11	3.6
	Seventh or more	26	8.6
Parents’ status			
	Living together at home	278	92.4
	Divorced	13	4.3
	Separated	1	0.3
	Widow	5	1.7
	Other	4	1.3
Total		304	100

**Figure 1:**
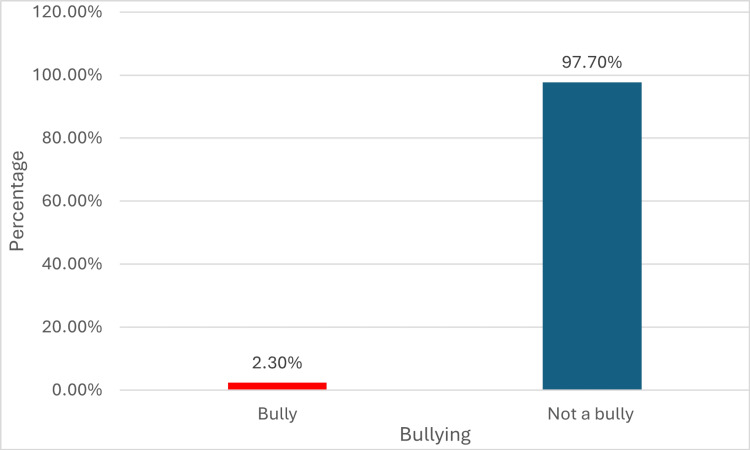
Bullying among female's middle school students in Riyadh

Moreover, this study examined bullying and bullying types. It was found that 2.3% (n=7) of the sample considered themselves bullies, as illustrated in Figure 1. Regarding bullying types, 3.8% (n=11) of the participants were verbally bullied. In terms of attitude toward bullying, the study used three categories: negative, neutral, and positive attitude; it appeared that 70.7% (n=215) of the sample had a neutral attitude toward bullying. When asking the students to assess their social environment, the results revealed that the majority of the participants had a good social environment.

**Table 2:** Relationship between bullying practice and social environment of female middle school students in Riyadh *p-value is highly significant < 0.01

Bullying practice	Social environment		Total	P-value by Fisher's exact test
	Good environment	Bad environment		
Not bully	188	3	191	0.000*
Bully	2	3	5	
Total	190	6	196	

Relationships

Table 2 represents the relationship between practising bullying and the social environment, which shows a highly significant association between the two variables (p=0.000). Table 3 demonstrates that there is a relationship between participants' attitudes toward bullying and their bullying practice (p = 0.0001). However, Table 4 suggests no correlation between students' attitude toward bullying and their social environment (p=0.756).

**Table 3:** Relationship between attitude toward bullying and practice bullying of female middle school students in Riyadh *p-value is significant < .05; degrees of freedom = 2; effect size = 0.25

Attitude toward bullying	Bullying practice		Total	P-value by chi-square test
	Bully	Not a bully		
Negative	2	64	66	0.0001*
Neutral	3	153	156	
Positive	2	4	6	
Total	7	221	228	

**Table 4:** The correlation between attitude toward bullying and social environment among female middle school students in Riyadh

Variable			Attitude toward bullying	Social environment
Spearman's rho	Attitude toward bullying	Correlation Coefficient	1.000	-0.024
		Sig. (2-tailed)	.	0.756
		N	249	175
	Social environment	Correlation Coefficient	-0.024	1.000
		Sig. (2-tailed)	0.756	.
		N	175	208

The results in Table 5 confirmed the change in students' attitudes toward bullying after implementing a health education program. It shows a highly significant difference in students' attitude toward bullying before and after the intervention (p=0.000, Z=-4.725).

**Table 5:** Change in female student’s attitude toward bullying after the Health Education Program *p value is highly significant < 0.01 a. post attitude < pre attitude b. post attitude > pre attitude c. post attitude = pre attitude

Variable		N	Mean rank	Sum of ranks	Z	P-value by Wilcoxon
Pre- and post-intervention attitude toward bullying	Negative ranks	79^a^	47.58	3758.50	-4.725^b^	0.000*
	Positive ranks	19^b^	57.50	1092.50		
	Ties	0^c^				
	Total	98				

## Discussion

This study attempts to assess students' bullying practices and attitudes toward bullying, identify the effect of the social environment and internal stimulation on bullying, implement a health education program, and then identify the intervention's effectiveness by reassessing students' attitudes.

Bullying

When asking students if they consider themselves to be practicing bullying, the majority revealed their disengagement from bullying behavior. This phenomenon could be attributed to the student's misconception of bullying, which is commonly referred to as joking and playing with peers in a rough way [19]. In this study, verbal bullying appeared as the most common type among the sample compared to the other types, which can be explained by the gender differences, suggesting that girls are more likely to be involved in verbal bullying [3]. Although the study found a low prevalence of self-reported bullying among participants, this result should be interpreted with caution. It is possible that underreporting occurred due to social desirability bias, where students may have responded in ways they believed were socially acceptable or favorable rather than truthful. Given the sensitive and stigmatized nature of bullying behavior, some students might have been reluctant to admit engaging in such actions, especially in a school setting.

Attitude toward bullying

The study's findings showed that more than half of the participants had a neutral attitude toward bullying. A recent study assumed that a neutral state "arises when positive and negative effects are absent or minimal"; in other words, people tend to identify as neutral when they have an average feeling, are unmotivated, or feel indifferent about the examined situation [20]. However, people express their attitude by using several terms, such as values, preferences, intentions, expectations, opinions, judgments, feelings, and beliefs [21]. When people are asked to make a judgment about an entity, they usually ask themselves, "How do I feel about it?" If their feelings are salient and work as a source of relevant information, judgments will be shaped. For illustration, if neutral affection is noticed and indicated indifference, this acts as relevant information that creates a neutral effect, leading to a neutral judgment [20].

Social cognitive theory elements and bullying 

Bullying and Social Environment

Bullying and social environments had a highly significant association in this study. This finding can be attributed to the fact that the majority of students reported not being bullies, in addition to having a positive and considerably safe social environment. A similar study that used SCT shows that the social environment has a clear impact on bullying; in the same way, these findings support the idea of SCT, which suggests that if a person sees aggression around them, they are more likely to bully others.

Bullying and Attitudes

The current study found a significant association between bullying and attitude toward bullying. Almost one-third of the participants had a negative attitude toward bullying, and concurrently, they were not practicing bullying; this can be explained by the SCT concept, which suggests a continuous interaction between the theory's elements [17]. This aligns with a study conducted in Northern California, which found that having a pro-bullying attitude can increase the likelihood of adopting bullying behavior and vice versa [22].

Attitude Toward Bullying and Social Environment

This study revealed no correlation between attitude toward bullying and the social environment, which contradicts the SCT that indicates the effect of the social environment in developing an attitude toward bullying [17]. However, the results may be explained by different factors that can affect developing an attitude other than the social environment, such as the person's moral judgments, which are the acceptability of the behavior [22].

The effectiveness of the Health Education Program

There was a highly significant difference between pre- and post-intervention attitudes toward bullying. A similar pattern of results was obtained in‏‏ a previous study, which found a significant difference in attitude toward bullying after the intervention; this can be due to the intervention's method, which mainly focused on engaging the audience in the program by asking for and arguing their opinions in different case scenarios, which was proven as an effective strategy for changing attitudes [23].

Limitations

One limitation of the health education program was its potential failure to adequately address the root causes of pro-bullying sentiments in Saudi Arabia. To effectively address bullying, it is advised that future interventions include developing an anti-bullying curriculum for schools, hosting frequent parent meetings to foster a secure social environment, and putting in place a thorough health education program that reaches all levels of the community. Further qualitative theory-based research would also be helpful in examining the elements influencing the nation's pro-bullying sentiments. The one-group pretest/post-test design, while practical, has methodological limitations that can affect the validity and generalizability of results. These include the absence of a control group, repeated exposure to the same questionnaire, selection bias, and the risk of researcher and participant biases. The study may also include participants with specific characteristics who were not representative of the general population. Additionally, reliance on self-reported data introduces the possibility of social desirability bias. These limitations weaken internal validity and limit the extent to which findings can be attributed to the intervention alone. The small sample size within certain subgroups, particularly those self-identified as bullies, reduces the statistical power to detect significant differences or draw reliable inferences.

## Conclusions

The findings indicate that most participants did not identify as bullies and had a neutral attitude toward bullying. The SCT conceptual elements revealed an association between bullying behavior and both attitudes toward bullying and the social environment. However, no significant relationship was observed between attitude and the social environment. Lastly, due to the effectiveness of the health education program, building a curriculum is necessary to promote an anti-bullying attitude in schools, besides carrying out further qualitative theory-based studies to explore the factors influencing pro-bullying attitudes in Saudi Arabia.

There is a need to build a school curriculum to reinforce anti-bullying attitudes and decrease tolerance toward bullying. Besides, it is necessary to establish regular meetings for students' parents with their teachers to promote a safe social environment and anti-bullying attitude. In addition, implementing a health education program targeting all community levels is a major requirement for tackling bullying. Further qualitative theory-based studies should be conducted to explore the factors under the pro-bullying attitude in Saudi Arabia. Future research should use a controlled experimental design, such as a randomized controlled trial, to accurately determine the causal impact of health education interventions on bullying attitudes and behaviours. Qualitative methods like focus groups or in-depth interviews can provide deeper insights into students' perceptions and motivations, enabling the design of interventions that are culturally and contextually appropriate. Longitudinal studies are also recommended to assess the long-term sustainability of attitude changes.
